# Supplementary material for: Bioaerosol generation by raindrops on soil
Source: Nat Commun. 2017 Mar 7;8:14668. doi: 10.1038/ncomms14668 (PMC5344306; doi:10.1038/ncomms14668)
Supplement: Supplementary Information — Supplementary Notes and Supplementary References [file ncomms14668-s1.pdf]

Supplementary Information

Supplementary Note 1

Estimation of bacteria transfer by raindrops from soil to air

First, we used the bacteria *aerosolization efficiency* range of 0.01 % to 0.001 %, as shown in Fig. 7d. It is known that the global bacteria surface density ranges from 10^4 cells/cm^2 to 10^8 cells/cm^2.[1] The total land area without ice is approximately 131×10^6 km^2, with 50 % of the soil containing clay.[2] Global average rainy days contributing 67 % of the total precipitation distributes up to 90 days.[3] To estimate, we used the middle range of rainy days, 45 days, similar to that of the Northwest United States.[3] Based on the average rainy days, we conservatively assumed that annually 45 raindrops hit on the same spot.

Supplementary References

1. Rastogi, R. *et al.* Comparative study of carbon nanotube dispersion using surfactants. *Journal of Colloid and Interface Science* **328**, 421-428, (2008).
2. Walther, J. V. *Earth's Natural Resources*. (Jones & Bartlett Learning, 2013).
3. Sun, Y., Solomon, S., Dai, A. & Portmann, R. W. How often does it rain? *Journal of Climate* **19**, 916-934, (2006).
